# Upregulation of microRNA-23b-3p induced by farnesoid X receptor regulates the proliferation and apoptosis of osteosarcoma cells

**DOI:** 10.1186/s13018-019-1404-6

**Published:** 2019-11-28

**Authors:** Bin Wu, Chengjuan Xing, Juan Tao

**Affiliations:** 1 Department of Thyroid Breast Surgery, Zhongshan Hospital Affiliated to Dalian University, Dalian, China; 2 Department of Pathology, Second Hospital Affiliated to Dalian Medical University, No.467 Zhongshan Road, Shahekou District, Dalian, 116027 Liaoning Province China

**Keywords:** Farnesoid X receptor, Osteosarcoma, Cyclin G1, Clone formation assay

## Abstract

**Background:**

The downstream targets of farnesoid X receptor (FXR) such as miRNAs have a potent effect on the progression of many types of cancer. We aim to study the effects of FXR on osteosarcoma (OS) development and the potential role of microRNA-23b-3p.

**Methods:**

The expressions of FXR and miR-23b-3p in normal osteoblasts and five osteosarcoma cell lines were measured. Their correlations were analyzed by Pearson’s test and verified by the introduction of FXR agonist, GW4064. TargetScan predicted that cyclin G1 (CCNG1) was a target for miR-23b-3p. The transfection of FXR siRNA was performed to confirm the correlation between FXR and miR-23b-3p. We further transfected miR-23b-3p inhibitor into MG-63 cells, and the transfected cells were treated with 5 μM GW4064 for 48 h. Quantitative PCR (qPCR) and Western blot were performed for expression analysis. Cell proliferation, cell apoptosis rate, and cell cycle distribution were assessed by clone formation assay and flow cytometry.

**Results:**

Scatter plot showed a positive correlation between FXR and miR-23b-3p (Pearson’s coefficient test *R*^2^ = 1.00, *P* = 0.0028). As CCNG1 is a target for miR-23b-3p, the treatment of GW4064 induced the downregulation of CCNG1 through upregulating miR-23b-3p. The inhibition of miR-23b-3p obviously promoted cell viability, proliferation, and cell cycle progression but reduced apoptosis rate of MG-63 cells; however, the treatment of GW4064 could partially reverse the effects of the inhibition of miR-23b-3p on OS cells.

**Conclusions:**

Upregulated FXR by GW4064 can obviously suppress OS cell development, and the suppressive effects may rely on miR-23b-3p/CCNG1 pathway.

## Background

Osteosarcoma (OS) is a frequent aggressive malignant bone tumor [1], and it is the second leading cause of cancer-related death among children and young adolescents [2]. The standard treatment for OS is surgery in combination with chemotherapy; however, OS has high recurrence and drug resistance and the 5-year survival rate is still less than 70% [3, 4]. Some recent studies revealed that the molecular pathogenesis of OS was highly associated with OS stem cells (OSCs) [5, 6]; however, the effective therapeutic targets and diagnostic markers for OS have not been identified yet [7]. Therefore, it is highly critical to better understand the OS pathology and the mechanism underlying the initiation and recurrence of the disease in order to develop strategies and new therapeutic methods in improving the prognosis of patients with osteosarcoma.

MicroRNAs (miRNAs) are a species of the non-protein coding RNA family, represented by short, single-stranded RNA approximately 18–22 nucleotides in length, and are the key regulators of post-transcriptional gene expression [8, 9]. Importantly, studies suggested a high association between abnormal miRNA expressions and the development and progression of numerous cancer types [9–12]. MiR-23b-3p, one of the many in miRNA family, has been considered as a biomarker for assisting the diagnosis and prognosis of different types of cancers such as ovarian cancer [13], prostate cancer [14], and hepatocellular carcinoma (HCC) [15]. In 2018, Xian et al. found that upregulating miR-23b-3p level could effectively inhibit gastric carcinoma cell proliferation, migration, and invasion through targeting cannabinoid receptor 1 (CB1R) [16]. Furthermore, miR-23b-3p could also inhibit cell proliferation in lung carcinoma by regulating the expression of cyclin G1 (CCNG1), which has been reported as an oncogene in OS development [17, 18]. These studies suggest that miR-23b-3p may also have a suppressive effect on OS cell proliferation by regulating CCNG1 expression.

Farnesoid X receptor (FXR, NR1H4), which serves as a ligand-activated transcription factor with multiple functions, is mainly expressed in the liver, intestine, kidneys, and adrenal glands [19, 20]. In the recent years, growing studies showed that the downstream targets of FXR such as miRNAs have a potent effect on multiple cancer progression, for example, Qiao et al. demonstrated that the upregulation of FXR by GW4064, an agonist of FXR, could significantly inhibit human colorectal cancer cell proliferation via miR-135A1/CCNG2 pathway [21]. In this study, we found a positive correlation between FXR expression and miR-23b-3p level. We also investigated whether FXR expression participated in OS development via miR-23b-3p/CCNG1 pathway.

## Methods

### Cell culture

Normal human osteoblasts (hFOB1.19) and osteosarcoma cell lines (MG-63, HOS, U2OS, SAOS2, and SJSA1) were obtained from American Type Culture Collection (ATCC, Manassas, VA, USA). All cell lines were cultured in Dulbecco’s modified Eagle’s medium (DMEM, Gibco, Thermo Fisher Scientific, Inc., Waltham, MA, USA) containing 10% fetal bovine serum (FBS; Gibco) and maintained in a 5% CO_2_ humidified incubator. The culture environment of normal osteoblasts was set at 34 °C, whereas osteosarcoma cells were cultured at 37 °C.

### Quantification of RNA

Total RNA including miRNAs was isolated using TRIzol (Invitrogen, CA, USA). The synthesis of cDNA was carried out using the RevertAid First Strand cDNA Synthesis kit (Thermo Fisher Scientific, Inc.) in a 10-μl reaction system. The reaction parameters were set at 65 °C for 5 min and then at 42 °C for 60 min. The relative mRNA levels were determined using the SYBR-Green PCR Master Mix kit (Takara, Dalian, China) on ABI 7500 real-time PCR system (Applied Biosystems, CA, USA). The thermocycling parameters were set at 95 °C for 30 s, followed by 40 cycles at 95 °C for 5 s and 60 °C for 34 s, and the primers for qPCR are listed in Table 1. The relative expression levels of miR-23b-3p and cellular genes were normalized to U6 and GAPDH, respectively. The result was calculated by 2^−ΔΔCt^ method [22].

**Table 1:** Primers for RT-qPCR

Gene name	Primer sequences
FXR	Forward: 5′-GATTGCTTTGCTGAAAGGGTC-3′
	Reverse: 5′- CAGAATGCCCAGACGGAAG-3′
miR-23b-3p	Forward: 5′-GAGCATCACATTGCCAGGG-3′
	Reverse: 5′-GTGCAGGGTCCGAGGT-3′
CCNG1	Forward: 5′- TTACCGCTGAGGAGCTGCAGTC-3′
	Reverse: 5′- CAGCCATCCTGGATGGATTCAG-3′
GAPDH	Forward: 5′- CACCGTCAAGGCTGAGAAC-3′
	Reverse: 5′- GGTGAAGACGCCAGTGGA-3′
U6	Forward: 5′-GCTTCGGCAGCACATATACTAAAAT-3′
	Reverse: 5′- CGCTTCACGAATTTGCGTGTCAT-3′

### Cell treatment

GW4064, a FXR agonist, was purchased from Sigma-Aldrich (#5172, Merck KGaA, Darmstadt, Germany). In order to study the correlation between FXR and miR-23b-3p, MG-63 cells were incubated with GW4064 at different concentrations. In the blank group, MG-63 cells were cultured in dimethyl sulfoxide vehicle, while in the GW4064 group, MG-63 cells were respectively treated by 0.5 and 5 μM GW4064. After 48 h of incubation, the cells were harvested for expression analysis.

### Luciferase reporter assay

TargetScan predicted that the sequence of CCNG1-3′-untranslated regions (UTR) contains a binding site of miR-23b-3p. PGL3-CCNG1 luciferase vector was constructed by cloning the fragment of CCNG1-3′-UTR, which contained putative binding sites for miR-23b-3p in the pGL3-Basic (Promega, Madison, WI, USA). HEK293T cells (ATCC) were plated onto 12-well plates (5 × 10^5^ cells/well) before the cell transfection. Subsequently, pGL-3 firefly luciferase reporters (1 μg per well) were respectively co-transfected with mimics control, miR-23b-3p mimics, and mutant (50 nM) using Lipofectamine 2000 reagent (Invitrogen). After 48 h of transfection, the luciferase activities were determined by dual-luciferase reporter assay system (Promega).

### Cell transfection

SiRNA for FXR (siFXR: 5′-CCUCAGGAAAUAACAAAUATT-3′), and non-specific scrambled siRNA (siNC: 5′-UUCUCCGAACGUGUCACGUTT-3′) were obtained from GenePharma (Shanghai GenePharma Co., Ltd., Shanghai, China). MG-63 cells at logarithmic phase were divided into four groups, and the cells were transfected by Lipofectamine 2000 (Invitrogen, Carlsbad, USA). In the siNC group, cells were transfected with siNC (50 nM). In siNC + GW4064 group, after transfection for 24 h, the cells were treated with GW4064 (5 μM) for 48 h. In the siFXR + GW4064 group, the cells with FXR knockdown were treated with GW4064 (5 μM) and incubated for 48 h. After the treatment, the cells were harvested for detecting transfection efficiencies by quantitative PCR (qPCR) and Western blot.

MiR-23b-3p inhibitor (anti-miR-23b-3p, 5′-UCUCAGUGCUUCUGGCUGC-3′) and inhibitor NC (anti-miR-NC, 5′-UCUACUCUUUCUAGGAGGUUGUGA-3′) were synthesized by GenePharma. The miR-23b-3p inhibitor and inhibitor control were transfected into MG-63 cells following the protocols of Lipofectamine 2000 (Invitrogen). The cells were assigned to four groups, namely, (A) IC group, in which MG-63 cells were transfected with inhibitor control; (B) IC + GW4064 group, in which cells were cultured with GW4064 (5 μM) for 48 h based on the IC group; (C) I group, in which MG-63 cells were transfected with miR-23b-3p inhibitor; and (D) I + GW4064 group, in which the transfected cells were treated with GW4064 (5 μM) for 48 h based on the I group. We further determine the levels of CCNG1 mRNA and protein to determine the transfection efficiencies of inhibitor control and miR-23b-3p inhibitor.

### Cell viability assay

Cell Counting Kit-8 (CCK-8, Dojindo, Kumamoto, Japan) was used for cell viability detection according to the manufacturer’s protocol. Cells from different treatment groups were seeded in a 96-well plate (5 × 10^3^ cells/well). CCK-8 reagent was added to each well to react and then incubated for 2 h. Absorbance value was determined using an enzyme microplate reader at 450 nm (Bio-Tek, Winooski, VT, USA).

### Clone formation assay

The ability of cell proliferation was assessed by soft agar clone formation assay as described previously [23]. Briefly, the cells (1 × 10^3^) were suspended in 1 ml DMEM containing 10% FBS and 0.3% low-melting agar (GE Healthcare, USA) on 6-well plates, which had been pre-coated by a solidified bottom layer made of 0.6% agarose in DMEM medium. After incubation for 2 weeks, cell culture was terminated when macroscopic apophyses were observed. The cells were fixed with 20% methanol for 15 min. Then, Giemsa solution was added to stain the cells for 40 min. After washing and air-drying, the clones were counted using a cloning counter.

### Annexin V/PI staining assay for apoptosis

Fluorescein isothiocyanate (FITC)-labeled annexin V (Annexin-V-FITC) apoptosis detection kit (Sigma-Aldrich) was performed to determine cell apoptosis on a flow cytometry (Gallios, Beckman Coulter, Inc., Brea, CA, USA). In brief, the transfected cells from each group were trypsinized and centrifuged at 1000×*g* for 5 min. Cells were washed twice by phosphate-buffered saline (PBS) and then incubated with 100 μL Annexin V-FITC and 5 μL propidium iodide (PI) in the dark for 15 min. The apoptosis rates were determined by flow cytometry.

### Cell cycle analysis

After 24 h of transfection, the transfected cells were cultured in 5 μM GW4064 and incubated for 48 h. Then, the cells were trypsinized and collected by centrifuging at 1000×*g* for 5 min. The cells were washed by PBS and fixed in 70% ethanol at 4 °C overnight and subsequently suspended in 400 μL buffer containing PI and RNase (BD Pharmingen, San Diego, CA, USA) for 30 min. The rates of cell cycle distribution were determined by flow cytometry (Gallios).

### Western blot analysis

Total proteins were extracted from the cells by RIPA protein extraction reagent (Thermo Fisher Scientific, Inc.). Then, cell lysate was subjected to 10% sodium lauryl sulfate-polyacrylamide gels (SDS-PAGE) and transferred onto polyvinylidene difluoride membranes (PVDF, Bio-Rad, Hercules, CA). After blocking the membranes by 5% non-fat milk for 1 h at room temperature, the membranes were cultured with primary antibodies. GAPDH (Mouse, #ab8245, 1:20000, 36 KD, Abcam, Cambridge, MA) served as an internal control. After incubation with secondary antibodies (goat anti-rabbit (1:20,000, 42 KD, #ab205718, Abcam), goat anti-mouse IgG (1:20,000, 52 KD, #ab205719, Abcam)) for 1 h at 4 °C, the specific signals were visualized by a LI-COR Odyssey Infrared Imaging System. Primary antibodies against CCNG1 (Mouse, 1:1000, #8016, 34 KD, Santa Cruz Biotechnology, Inc., Dallas, USA), FXR (Mouse, 1:1000, #25309, 70 KD, Santa Cruz Biotechnology, Inc.), B-cell lymphoma-2 (Bcl-2, Rabbit, #ab59348, 1:1000, 26 KD Abcam), Bax (Rabbit, #ab32503, 1:2000, 21 KD, Abcam), and Cleaved Caspase-3 (Rabbit, #ab2302, 1:1000, 17 KD, Abcam) were used.

### Statistical analyses

All data were expressed as the mean ± SEM. Pearson’s correlation coefficient (PCC) was determined in Microsoft Excel according to the expressions of FXR and miR-23b-3p. Student’s *t* test was used for evaluating the difference between two group values, and ANOVA analysis followed by Dunnett’s *t* test was used for determining statistical significance of multiple groups. *P* < 0.05 was considered as statistically significant.

## Results

### Correlation of FXR and miR-23b-3p expression in OS cells

**Fig. 1:**
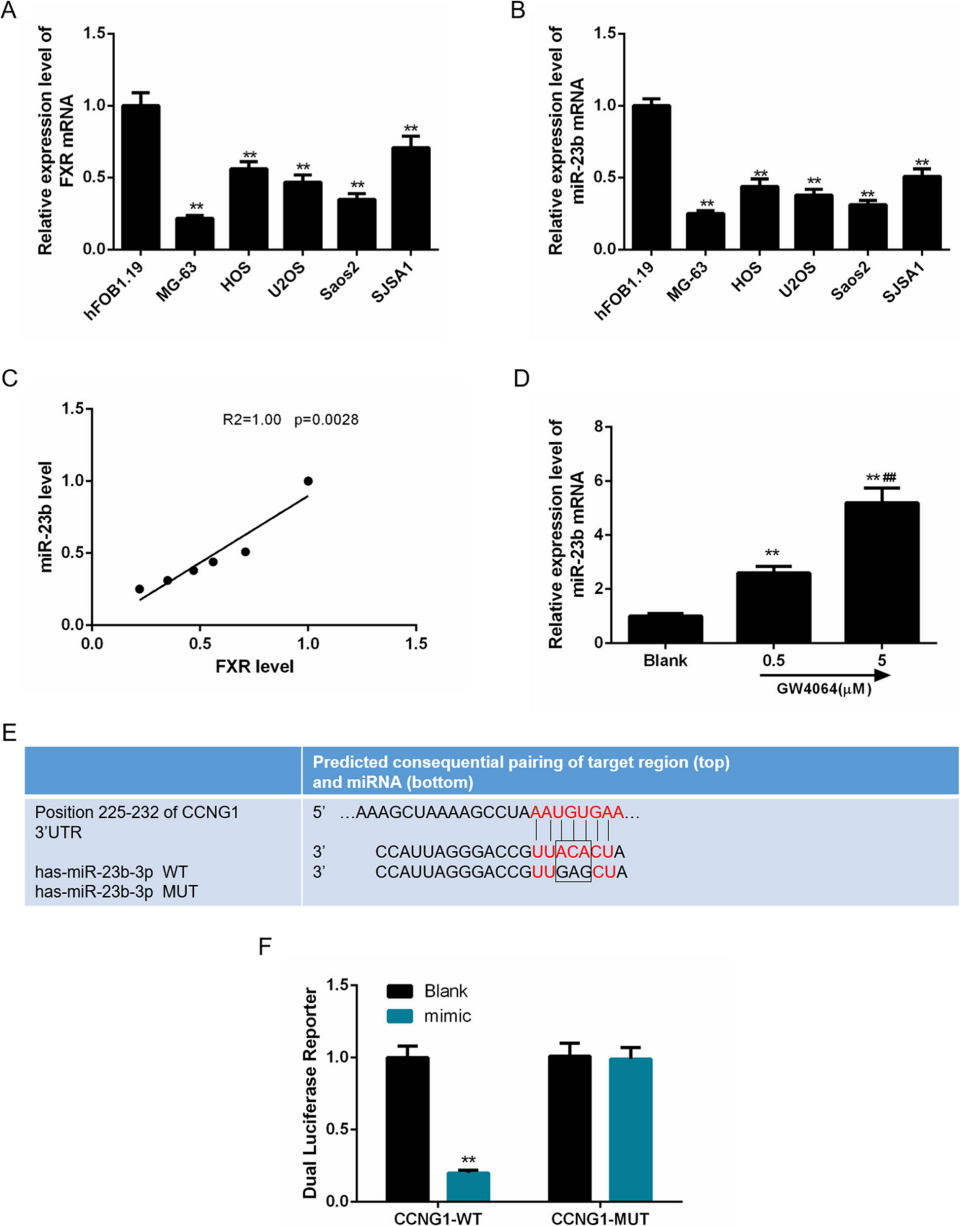
Correlation of farnesoid X receptor (FXR) and microRNA-23b-3p expression in osteosarcoma (OS) cells and miR-23b-3p specifically targets cyclin G1 (CCNG1). As FXR might regulate the expression of miR-23b-3p in OS cells, we measured the expressions of FXR and miR-23b-3p in normal osteoblasts (hFOB1.19) and five osteosarcoma cell lines (MG-63, HOS, U2OS, SAOS2, SJSA1). **a** The relative expression levels of FXR were much downregulated in OS cell lines, especially in MG-63 cells. **b** The expressions of miR-23b-3p were obviously decreased, especially in MG-63 cells compared with hFOB1.19 cells. **c** Scatter plots showed the correlation of FXR and miR-23b-3p expression in normal osteoblasts and five osteosarcoma cell lines (Pearson’s coefficient test *R*^2^ = 1.00, *P* = 0.0028). **d** The changes of miR-23b-3p levels were measured under different concentrations of the FXR agonist, GE4064 (0, 0.5, and 5 μM). **e** TargetScan predicted that the fragment of CCNG1-3′-UTR contained a binding site of miR-23b-3p. **f** The correlation between miR-23b-3p and CCNG1 was verified by luciferase reporter assay. Each value represents mean ± SEM (*n* = 3). GAPDH and U6 served as internal controls for cellular genes and miR-23b-3p, respectively. **p < 0.01 vs. hFOB1.19 cells or blank group; ^##^*p* < 0.01 vs. 0.5 μM GW4064 group

As FXR might regulate miR-23b-3p expression in OS cells, we compared the expression level of FXR with that of miR-23b-3p in hFOB1.19 cells and five types of OS cell lines (MG-63, HOS, U2OS, SAOS2, and SJSA1). In Fig. 1 a and b, in comparison with that in hFOB1.19 cells, relative expression levels of FXR and miR-23b-3p in OS cell lines were significantly downregulated and were the lowest in MG-63 cells, meanwhile, the scatter plot showed a positive correlation between BAG3 and IL-8 (Pearson’s coefficient test *R*^2^ = 1, *P* = 0.0028, Fig. 1c). Furthermore, for the correlation between FXR and miR-23b-3p, 0.5 and 5 μM FXR agonist GE4064 were used to treat MG-63 cells, and we observed that the miR-23b-3p level was obviously upregulated with the increased concentrations of GW4064, indicating that FXR could positively regulate miR-23b-3p expression (*p* < 0.01, Fig. 1d). Collectively, our findings suggest that miR-23b-3p levels are positively associated with FXR expression levels in OS cells.

### MiR-23b-3p specifically targets CCNG1

TargetScan predicted that the position of 225–232 of CCNG1-3′-UTR contained a binding site of miR-23b-3p (Fig. 1e, f). After the verification of luciferase reporter assay, the luciferase activity of cells co-transfected with PGL3-CCNG1 luciferase and miR-23b-3p WT vectors was found much lower than the blank group (*p* < 0.01), indicating that miR-23b-3p could effectively bind to CCNG1-3′-UTR. Meanwhile, no difference was observed between the luciferase activity of cells co-transfected with PGL3-CCNG1 luciferase and miR-23b-3p MUT vectors and that of the blank group. Thus, CCNG1 could be seen as a promising target for miR-23b-3p.

### GW4064 treatment indirectly regulates CCNG1 expression via miR-23b-3p

**Fig. 2:**
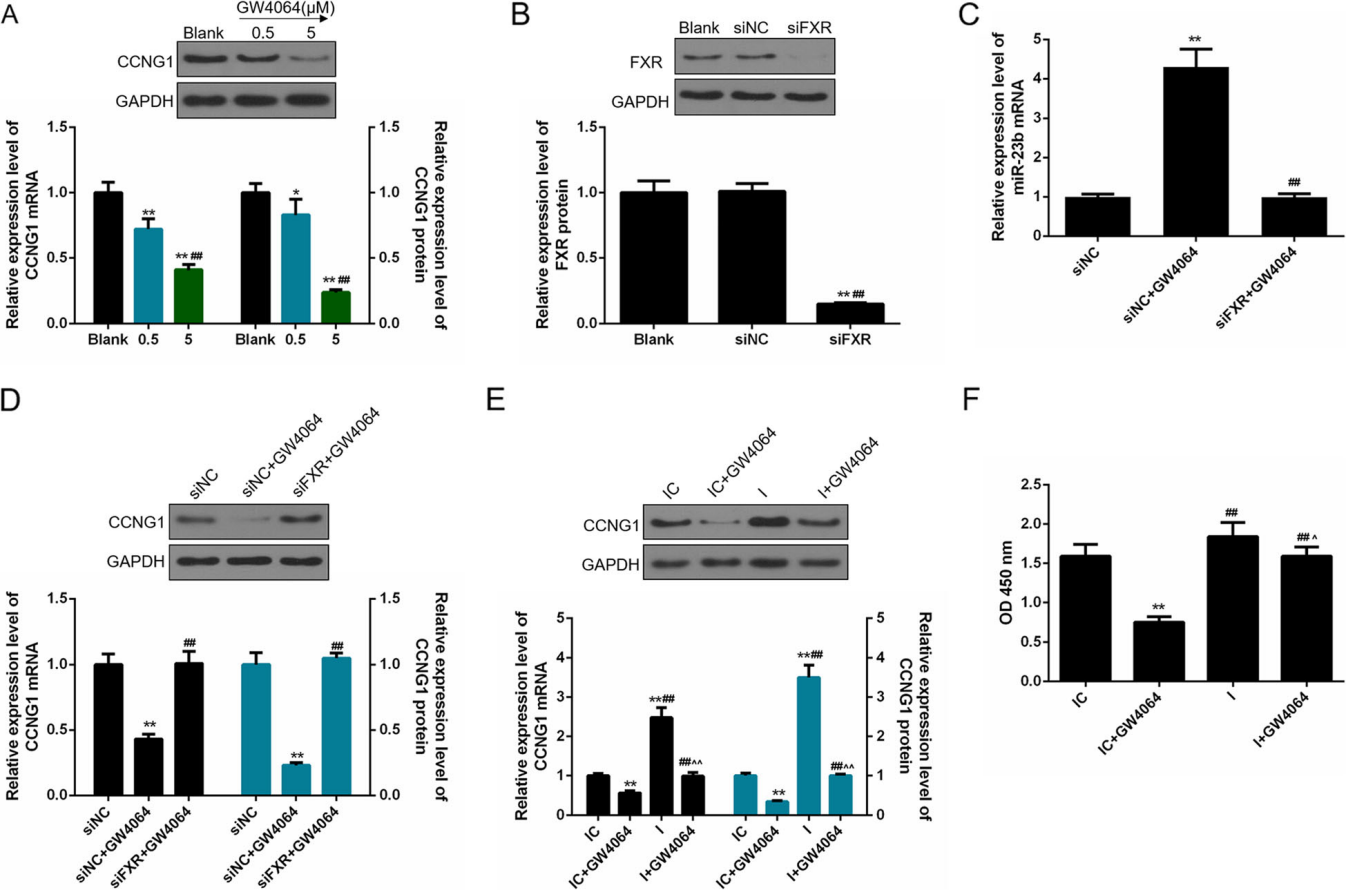
GW4064 treatment indirectly regulated cyclin G1 (CCNG1) expression via microRNA-23b-3p. CCNG1 was identified as a promising target of miR-23b-3p. Considering the positive correlation between farnesoid X receptor (FXR) and miR-23b-3p, we further assessed the correlation between FXR and CCNG1 expression. **a** The changes of CCNG1 mRNA and protein levels were measured by quantitative PCR (qPCR) and Western blot under the effects of increased concentrations of GW4064. **b** The transfection efficiency of FXR siRNA (siFXR) was measured by qPCR and Western blot. **c** The combined effect of siFXR and GW4064 on the expression of miR-23b-3p was examined by qPCR. **d** The combined effect of siFXR and GW4064 on CCNG1 expressions was measured by qPCR and Western blot. **e** We further transfected miR-23b-3p inhibitor and inhibitor control into MG-63 cells. The single and combined effects of inhibiting miR-23b-3p and GW4064 effects on CCNG1 were detected by qPCR and Western blot. **f** The changes of MG-63 cell viability were determined by Cell Counting Kit-8 (CCK-8) assay. Each value represents mean ± SEM (*n* = 3). GAPDH and U6 served as internal controls for cellular genes and miR-23b-3p, respectively. **p < 0.01 vs. blank (siNC or IC) group; ^##^*p* < 0.01 vs. siNC + GW4064 group; ^*p* < 0.05, ^^*p* < 0.01 vs. I group

We have identified that CCNG1 is a potential target for miR-23b-3p. As there is a positive correlation between FXR and miR-23b-3p, we were interested in investigating whether FXR could indirectly regulate CCNG1 expression via miR-23b-3p. According to the results in Fig. 2a, with the increased concentrations of GW4064, the mRNA and protein levels of CCNG1 were both gradually downregulated and were then lowest under the effects of 5 μM CW4064 treatment (*p* < 0.01). In order to further verify the negative correlation between FXR and CCNG1 expressions, siFXR was transfected into MG-63 cells (Fig. 2b). The transfection of siFXR could effectively inhibit upregulated miR-23b-3p induced by GW4064 (*p* < 0.01, Fig. 2c) and substantially enhanced the expression of CCNG1 (*p* < 0.01, Fig. 2d).

In order to confirm whether the negative regulatory effects of FXR on CCNG1 expression were mediated through the regulation of miR-23b-3p, miR-23b-3p inhibitor and inhibitor control were transfected into MG-63 cells. As shown in Fig. 2e, both the levels of CCNG1 mRNA and protein were obviously downregulated under the treatment of GW4064 and peaked in the cells transfected miR-23b-3p inhibitor without GW4064 treatment (*p* < 0.01). When the blockage of miR-23b-3p of cells was treated with GW4064, the CCNG1 expressions were greatly downregulated (*p* < 0.01). We additionally measured the changes in the cell viability and observed that MG-63 cell viability was obviously decreased in the IC + GW4064 group (*p* < 0.01) but reached a peak in the I group. After the treatment of GW4064, the enhanced cell viability by miR-23b-3p inhibitor was decreased significantly again (*p* < 0.05, Fig. 2f). Together, the upregulated FXR expression by GW4064 could promote miR-23b-3p expression, which could subsequently inhibit CCNG1 expression.

### GW4064 treatment suppresses cell proliferation and promoted apoptosis and cell cycle arrest in MG-63 cells

**Fig. 3:**
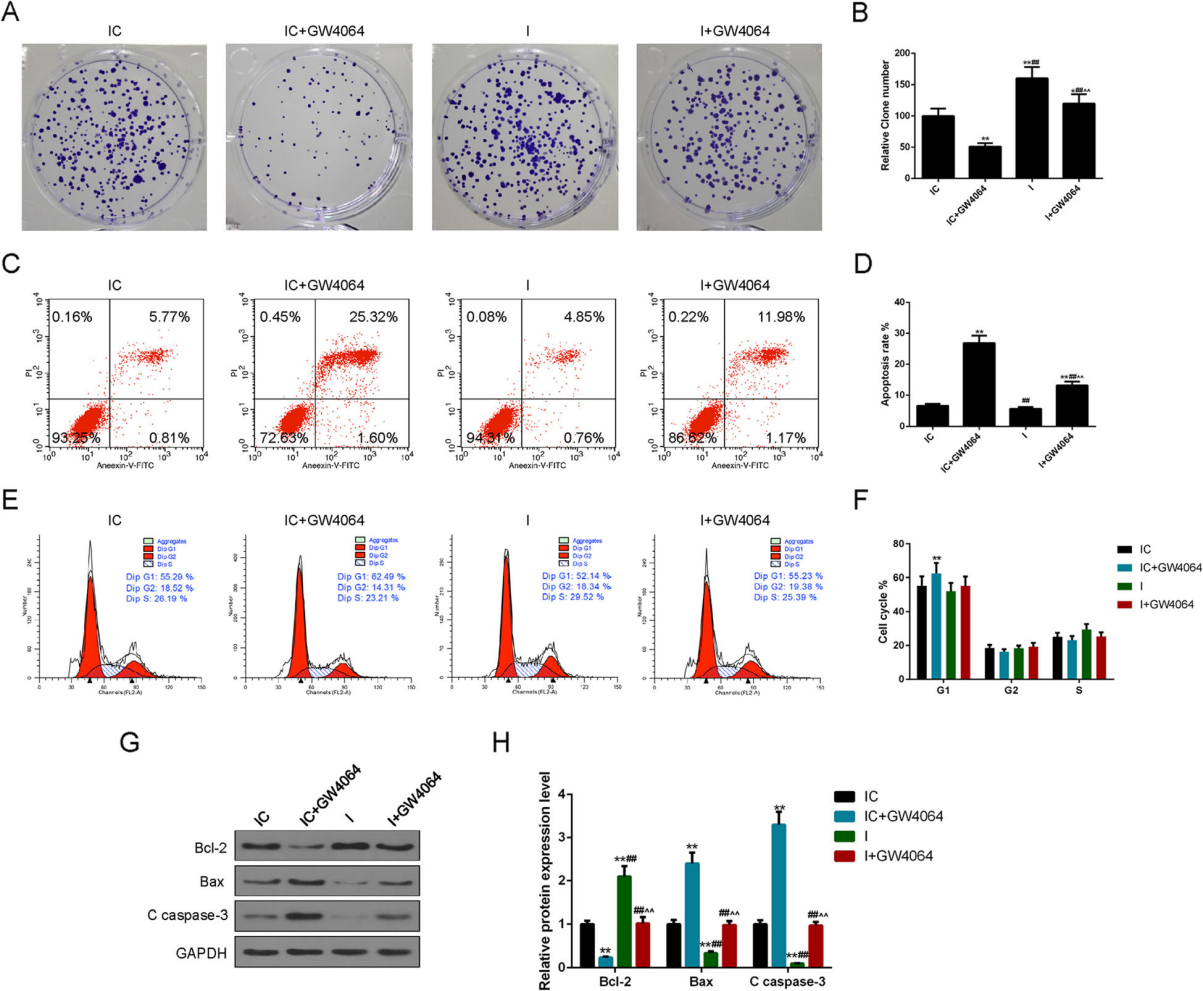
GW4064 treatment inhibited cell proliferation and induced apoptosis and cell cycle arrest in MG-63 cells. We further study the effects of farnesoid X receptor (FXR) regulation on the development of MG-63 cells and the role of microRNA-23b-3p during the progress. **a**, **b** In order to determine the effects of FXR regulation on osteosarcoma (OS) cell proliferation, clone formation assay of MG-63 cells were performed under the single and combined effects of miR-23b-3p inhibition and GW4064 treatment. **c**, **d** The changes of apoptosis rate were analyzed by flow cytometry. **e**, **f** The cell cycle distribution of MG-63 cells was determined by flow cytometry. **g**, **h** The protein levels of several apoptotic genes (B-cell lymphoma-2 (Bcl-2), Bax, and Cleaved Caspase-3 (C caspase-3)) were analyzed by Western blot to further assess the effects of FXR regulation on OS cell apoptosis. Each value represents mean ± SEM (*n* = 3). GAPDH served as an internal control. *p < 0.05, **p < 0.01 vs. IC group; ^##^*p* < 0.01 vs. IC + GW4064 group; ^^*p* < 0.01 vs. I group

We further study the functions of FXR in the development of OS. Clone formation assay showed that the inhibition of miR-23b-3p obviously promoted MG-63 cell proliferation, while GW4064 treatment significantly inhibited the enhanced OS cell proliferation (*p* < 0.05, Fig. 3a, b). Cell apoptosis and cell cycle distribution were determined by flow cytometry, as shown in Fig. 3 c and d; GW4064 treatment significantly increased the apoptosis rate from 6.58% of the IC group to 26.92% of the IC + GW4064 group (*p* < 0.01), while the inhibition of mir-23b-3p greatly inhibited MG-63 cell apoptosis; meanwhile, GW4064 treatment also obviously increased apoptosis rate of cells transfected with mir-23b-3p inhibitor (I + GW4064 vs. I, 13.15% vs. 5.61%, *p* < 0.01). The distribution of cell cycle is presented in Fig. 3 e and f; it could be found that GW4064 treatment greatly increased the rate of G1 phase from 55.29% of the IC group to 62.49% of the IC + GW4064 group, indicating that enhancing FXR may contribute to the induced cell cycle arrest at G1 phase while decreased rate of G1 phase and increased rate of S phase in the I group indicated a promoting effect of the inhibition of mir-23b-3p on cell cycle progression. We also observed that the decreased rate of G1 phase by mir-23b-3p inhibitor was increased again after the treatment of GW4064 (52.14% vs. 55.23%).

The protein levels of Bcl-2, Bax, and C caspase-3 were key indicators of cell apoptosis (Fig. 3g, h). In the study, GW4064 treatment obviously inhibited Bcl-2 protein level but increased Bax and C caspase-3 levels (*p* < 0.01), while the mir-23b-3p inhibitor significantly promoted Bcl-2 expression and inhibit Bax and C caspase-3 levels (*p* < 0.01). Importantly, the treatment of GW4064 could partially reverse the effects of mir-23b-3p inhibitor on Bcl-2, Bax, and C caspase-3 protein levels. Taken together, our data indicated that the upregulation of FXR could induce cell cycle arrest and promote apoptosis in OS.

## Discussion

We observed that both FXR and miR-23b-3p expressions decreased observably in OS cell lines, compared with normal osteoblasts (hFOB1.19) cells. Then, by treating the cells with FXR agonist GW4064, we confirmed a positive correlation between the expressions of FXR and miR-23b-3p in human OS cells. CCNG1 was confirmed as a target for miR-23b-3p, and FXR participated in the regulation of CCNG1 via miR-23b-3p. In addition, we also observed that the upregulated FXR by GW4064 could reduce cell proliferation, enhance apoptosis, and induce cell cycle arrest in OS cells. Therefore, based on the positive correlation between FXR and miR-23b-3p, the anti-cancer effects of FXR on OS cell development were associated with the regulation of miR-23b-3p/CCNG1 pathway.

Growing studies have reported that miR-23b-3p functions as a predictor and prognostic molecular marker for different cancer types such as esophageal squamous cell carcinomas [17], pancreatic ductal adenocarcinoma [24], and HCC [15]. In our study, we found a positive correlation between miR-23b-3p and FXR and that GW4064 treatment could induce the upregulation of FXR and miR-23b-3p. Therefore, we reasonably speculated that miR-23b-3p was involved in the upregulation of FXR by GW4064 in suppressing cell proliferation and promoting apoptosis and cell cycle arrest in OS. These data are consistent with previous researches, in which enhancing miR-23b-3p expression mediated several targets to effectively affect tumor cell development, proliferation, and metastasis, and ultimately inhibited cancer development and progression [13, 25, 26].

The regulatory effects of miRNAs on the progression of cancer largely rely on directly regulating the expressions of their target genes. Previous study has revealed that miR-23b-3p specifically targets CCNG1 to regulate HCC cell proliferation and invasion [17]. We also confirmed the close relationship between CCNG1 and miR-23b-3p. CCNG1 is a pivotal component of commanding cyclin G1/murine double minute gene 2/tumor protein p53 (CCNG1/Mdm2/p53) axis [27]. P53 functions as a key regulator of cell cycle progression [28]. CCNG1 could stimulate and propel PP2A catalytic activity toward E3 ubiquitin-protein ligase (Mdm2), which has increased or abnormal expression in numerous cancer types including OS [29, 30]. Then, activation of Mdm2 antagonizes the p53 tumor suppressor function by forming a physical complex with p53 [27]. Previous studies have demonstrated the promoting effects of CCNG1 on cell growth and that the inhibition of CCNG1 could effectively suppress the growth of human tumor xenografts in a nude mouse model [18, 31]. Currently, the potential of companion diagnostics of CCNG1 pathway in the staging, prognosis, and treatment of cancers is widely recognized. In the current study, our data showed that FXR could negatively regulate the expression of CCNG through miR-23b-3p, indicating that the inhibitory effects of FXR on OS cell proliferation and growth were highly associated with the downregulated CCNG1 level.

In recent years, FXR has attracted increasing attention as a therapeutic target in the treatment of cancers [32]. Data from previous reports indicated that FXR deficiency could induce the formation of spontaneous liver tumors and small intestine adenocarcinoma in APCmin mice and promote colon cell proliferation [33, 34]. These studies showed that FXR plays a tumor-suppressive role in certain cancers [35, 36]. In our study, the expressions of FXR in the OS cell lines were also generally lower than that of normal osteoblasts, while the upregulation of FXR by GW4064 showed a potent effect on OS cell proliferation, apoptosis, and cell cycle distribution. The downstream factors of FXR, including miRNAs, have been proved to participate in multiple carcinogenesis [37]. In 2013, Song et al. demonstrated a novel role of peroxisome proliferator-activated receptor gamma (PPARγ), that is, it could epigenetically induce the upregulation of miRNA transcription [38]. FXR shares a number of characteristics with PPARγ and it could bind to retinoid X receptor α (RXRα) as a monomer, and subsequently recruits different cofactors to induce the expressions of target genes in HCC cells [32, 39]. However, whether FXR can epigenetically induce miR-23b-3p expression as PPARγ is still unclear; however, this may be partially explained by the positive correlation between miR-23b-3p and FXR; however, the molecular mechanism requires further study. It should be noted that in vivo model was not constructed in this study, which was a limitation in this study.

## Conclusion

In conclusion, our data observed that both FXR and miR-23b-3p were downregulated in OS cells. Pearson’s test demonstrated a positive correlation between miR-23b-3p and FXR, and the positive correlation was further verified by the introduction of GW4064. Furthermore, the upregulation of FXR by GW4064 can suppress OS cell proliferation and promote apoptosis and cell cycle arrest, while inhibiting miR-23b-3p has a promoting effect on OS cell development. CCNG1 is a target for miR-23b-3p, indicating that the tumor-suppressive effects of FXR may be highly associated with miR-23b-3p/CCNG1 pathway. These results showed that FXR/miR-135A1/CCNG2 axis is possibly a potential therapeutic target for OS.

## Data Availability

The analyzed data sets generated during the study are available from the corresponding author on reasonable request.

## Abbreviations

Annexin-V-FITC: FITC-labeled annexin V
ATCC: American Type Culture Collection
Bcl-2: B-cell lymphoma-2
C caspase-3: Cleaved Caspase-3
CB1R: Cannabinoid receptor 1
CCK-8: Cell Counting Kit-8
CCNG1: Cyclin G1
CCNG1/Mdm2/p53: Cyclin G1/murine double minute gene 2/tumor protein p53
DMEM: Dulbecco’s modified Eagle’s medium
FBS: Fetal bovine serum
FITC: Fluorescein isothiocyanate
FXR: Farnesoid X Receptor
HCC: Hepatocellular carcinoma
Mdm2: E3 ubiquitin-protein ligase
miRNAs: MicroRNAs
OS: Osteosarcoma
OSCs: OS stem cells
PBS: Phosphate-buffered saline
PCC: Pearson’s correlation coefficient
PI: Propidium iodide
PPARγ: Peroxisome proliferator-activated receptor gamma
PVDF: Polyvinylidene difluoride membranes
qPCR: Quantitative PCR
RXRα: Retinoid X receptor α
SDS-PAGE: Sodium lauryl sulfate-polyacrylamide gels
siFXR: FXR siRNA
UTR: Untranslated regions

## References

[1] Moore DD, Luu HH (2014). Osteosarcoma. Cancer Treat Res.

[2] Yan GN, Lv YF, Guo QN (2016). Advances in osteosarcoma stem cell research and opportunities for novel therapeutic targets. Cancer Lett.

[3] Bielack SS, Kempf-Bielack B, Delling G, Exner GU, Flege S, Helmke K (2002). Prognostic factors in high-grade osteosarcoma of the extremities or trunk: an analysis of 1,702 patients treated on neoadjuvant cooperative osteosarcoma study group protocols. J Clin Oncol.

[4] Ta HT, Dass CR, Choong PF, Dunstan DE (2009). Osteosarcoma treatment: state of the art. Cancer Metastasis Rev.

[5] He JP, Hao Y, Wang XL, Yang XJ, Shao JF, Guo FJ (2014). Review of the molecular pathogenesis of osteosarcoma. Asian Pac J Cancer Prev.

[6] Otoukesh B, Boddouhi B, Moghtadaei M, Kaghazian P, Kaghazian M (2018). Novel molecular insights and new therapeutic strategies in osteosarcoma. Cancer Cell Int.

[7] Liu G, Huang K, Jie Z, Wu Y, Chen J, Chen Z (2018). CircFAT1 sponges miR-375 to promote the expression of Yes-associated protein 1 in osteosarcoma cells. Mol Cancer.

[8] Cui Z, Liu G, Kong D (2018). miRNA27a promotes the proliferation and inhibits apoptosis of human pancreatic cancer cells by Wnt/beta-catenin pathway. Oncol Rep.

[9] Lu Y, Tang L, Zhang Z, Li S, Liang S, Ji L (2018). Long noncoding RNA TUG1/miR-29c axis affects cell proliferation, invasion, and migration in human pancreatic cancer. Dis Markers.

[10] Noori J, Sharifi M, Haghjooy JS (2018). miR-30a inhibits melanoma tumor metastasis by targeting the E-cadherin and zinc finger E-box binding homeobox 2. Adv Biomed Res.

[11] Liu S, Chen J, Zhang T and Chen H. MicroRNA-133 inhibits the growth and metastasis of the human lung cancer cells by targeting epidermal growth factor receptor. J buon. 2019;24:929–35. 31424644

[12] Zhou W, Hao M, Du X, Chen K, Wang G, Yang J (2014). Advances in targeted therapy for osteosarcoma. Discov Med.

[13] Su L, Liu M (2018). Correlation analysis on the expression levels of microRNA-23a and microRNA-23b and the incidence and prognosis of ovarian cancer. Oncol Lett.

[14] Li D, Hao X, Song Y (2018). Identification of the key MicroRNAs and the miRNA-mRNA regulatory pathways in prostate cancer by bioinformatics methods. Biomed Res Int.

[15] Jiang T, Huang Z, Zhang S, Zou W, Xiang L, Wu X (2018). miR23b inhibits proliferation of SMMC7721 cells by directly targeting IL11. Mol Med Rep.

[16] Xian X, Tang L, Wu C, Huang L (2018). miR-23b-3p and miR-130a-5p affect cell growth, migration and invasion by targeting CB1R via the Wnt/β-catenin signaling pathway in gastric carcinoma. Onco Targets Ther..

[17] Zhang J, Zhang Y, Tan X, Zhang Q, Liu C, Zhang Y (2018). MiR-23b-3p induces the proliferation and metastasis of esophageal squamous cell carcinomas cells through the inhibition of EBF3. Acta Biochim Biophys Sin Shanghai.

[18] Skotzko M, Wu L, Anderson WF, Gordon EM, Hall FL (1995). Retroviral vector-mediated gene transfer of antisense cyclin G1 (CYCG1) inhibits proliferation of human osteogenic sarcoma cells. Cancer Res.

[19] Calkin AC, Tontonoz P (2012). Transcriptional integration of metabolism by the nuclear sterol-activated receptors LXR and FXR. Nat Rev Mol Cell Biol.

[20] Forman BM, Goode E, Chen J, Oro AE, Bradley DJ, Perlmann T (1995). Identification of a nuclear receptor that is activated by farnesol metabolites. Cell.

[21] Qiao P, Li S, Zhang H, Yao L, Wang F (2018). Farnesoid X receptor inhibits proliferation of human colorectal cancer cells via the miR135A1/CCNG2 signaling pathway. Oncol Rep.

[22] Livak KJ, Schmittgen TD (2001). Analysis of relative gene expression data using real-time quantitative PCR and the 2(-Delta Delta C(T)) Method. Methods.

[23] Ye M, Li J, Gong J (2017). PCDH10 gene inhibits cell proliferation and induces cell apoptosis by inhibiting the PI3K/Akt signaling pathway in hepatocellular carcinoma cells. Oncol Rep.

[24] Wei DM, Dang YW, Feng ZB, Liang L, Zhang L, Tang RX (2018). Biological effect and mechanism of the miR-23b-3p/ANXA2 axis in pancreatic ductal adenocarcinoma. Cell Physiol Biochem.

[25] YiRen H, YingCong Y, Sunwu Y, Keqin L, Xiaochun T, Senrui C (2017). Long noncoding RNA MALAT1 regulates autophagy associated chemoresistance via miR-23b-3p sequestration in gastric cancer. Mol Cancer.

[26] Xian X, Tang L, Wu C, Huang L (2018). miR-23b-3p and miR-130a-5p affect cell growth, migration and invasion by targeting CB1R via the Wnt/beta-catenin signaling pathway in gastric carcinoma. Onco Targets Ther.

[27] Al-Shihabi A, Chawla SP, Hall FL, Gordon EM (2018). Exploiting oncogenic drivers along the CCNG1 pathway for cancer therapy and gene therapy. Mol Ther Oncolytics.

[28] Gordon EM, Ravicz JR, Liu S, Chawla SP, Hall FL (2018). Cell cycle checkpoint control: the cyclin G1/Mdm2/p53 axis emerges as a strategic target for broad-spectrum cancer gene therapy - a review of molecular mechanisms for oncologists. Mol Clin Oncol.

[29] Piette J, Neel H, Marechal V (1997). Mdm2: keeping p53 under control. Oncogene.

[30] Momand J, Jung D, Wilczynski S, Niland J (1998). The MDM2 gene amplification database. Nucleic Acids Res.

[31] Chen DS, Zhu NL, Hung G, Skotzko MJ, Hinton DR, Tolo V (1997). Retroviral vector-mediated transfer of an antisense cyclin G1 construct inhibits osteosarcoma tumor growth in nude mice. Hum Gene Ther.

[32] He J, Zhao K, Zheng L, Xu Z, Gong W, Chen S (2015). Upregulation of microRNA-122 by farnesoid X receptor suppresses the growth of hepatocellular carcinoma cells. Mol Cancer.

[33] Yang F, Huang X, Yi T, Yen Y, Moore DD, Huang W (2007). Spontaneous development of liver tumors in the absence of the bile acid receptor farnesoid X receptor. Cancer Res.

[34] Maran RR, Thomas A, Roth M, Sheng Z, Esterly N, Pinson D (2009). Farnesoid X receptor deficiency in mice leads to increased intestinal epithelial cell proliferation and tumor development. J Pharmacol Exp Ther.

[35] Bailey AM, Zhan L, Maru D, Shureiqi I, Pickering CR, Kiriakova G (2014). FXR silencing in human colon cancer by DNA methylation and KRAS signaling. Am J Physiol Gastrointest Liver Physiol.

[36] Gadaleta RM, Cariello M, Sabbà C, Moschetta A (2015). Tissue-specific actions of FXR in metabolism and cancer. Biochim Biophys Acta.

[37] Yang F, Gong J, Wang G, Chen P, Yang L, Wang Z (2016). Waltonitone inhibits proliferation of hepatoma cells and tumorigenesis via FXR-miR-22-CCNA2 signaling pathway. Oncotarget.

[38] Song K, Han C, Zhang J, Lu D, Dash S, Feitelson M (2013). Epigenetic regulation of MicroRNA-122 by peroxisome proliferator activated receptor-gamma and hepatitis b virus X protein in hepatocellular carcinoma cells. Hepatology.

[39] de Aguiar Vallim TQ, Tarling EJ, Kim T, Civelek M, Baldan A, Esau C (2013). MicroRNA-144 regulates hepatic ATP binding cassette transporter A1 and plasma high-density lipoprotein after activation of the nuclear receptor farnesoid X receptor. Circ Res.

